# The Terpenoid Alkaloids of Colobognath Millipedes: Insights into Structural Diversity and Function

**DOI:** 10.1021/acs.jnatprod.5c01363

**Published:** 2026-01-09

**Authors:** Arden Hatch, Paul Marek, Emily Mevers

**Affiliations:** † Department of Chemistry, 1757 Virginia Tech, Blacksburg, Virginia 24061, United States; ‡ Department of Entomology, 1757 Virginia Tech, Blacksburg, Virginia 24061, United States

## Abstract

Colobognatha, a group of millipedes (Diplopoda) known for their unique biological traits (*e.g.,* brood care and sociality), is the only group among millipedes to produce terpenoid alkaloids. Before 2020, only four terpenoid alkaloids had been identified; however, recent studies have resulted in a surge of new chemical discoveries and research into their ecological and biochemical roles. In this review, we outline the social characteristics of Colobognatha, the chemical investigations of their defensive secretions, and the bioactivity of the terpenoid alkaloids with a particular emphasis on new findings. We conclude by summarizing gaps in the research on these chemicals and provide insights into future research directions.

## Graphical abstract

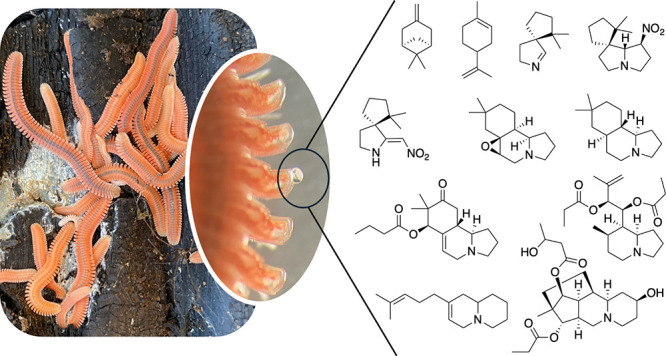

## Introduction

Millipedes (Diplopoda) are a diverse and ancient class of arthropods, representing one of the oldest groups of terrestrial animals, with early fossils from over 425 million years ago (mya).^1,2^ To date, about 13,000 millipede species have been described, but it is estimated that there are closer to 70,000 species, with most still undescribed—primarily in the Tropical Region.^3^ Primitive defenses against predators included mechanical means such as spines, armor, and the ability to roll into an armadillo-like ball, but later in the evolutionary history of the group, chemical defenses evolved.^4^ Extant millipedes are known to produce a wide variety of defensive chemicals, including hydrogen cyanide, oxidized aromatics (*e.g.,* benzoquinones), and alkaloids (*e.g.,* quinazolinone and terpene alkaloids).^4–8^ These compounds are stored in high concentrations in ozadenes/glands (or rapidly generated from inert biological precursors), and the millipedes release these defense agents through specialized openings of the glands called ozopores when disturbed.^9,10^ This chemical defense mechanism evolved a very long time ago, as suggested by a 385-mya Devonian fossilized millipede showing the presence of these ozopores lining the body.^11^ The chemical is not known, but was likely an oxidized aromatic due to the identity of the fossil in the superorder Juliformia. As millipedes have evolved and diversified, so too have their chemical defenses. Hydrogen cyanide is produced by one species-rich order (Polydesmida), oxidized aromatics are produced by six orders (Juliformia and Nematophora), quinazolinone alkaloids are produced by one order (Glomerida), and the terpenoid alkaloids are produced by four orders (Colobognatha).^4^ There is only minimal overlap of chemicals among these groups. The defensive secretion composition of all millipedes has been reviewed previously,^4,9^ in 1978 and 2015. However, since the most recent review, there have been many significant new findings on the terpenoid alkaloids that greatly expand the known defensive secretions and their ecological role within species. Within Colobognatha, there is considerable diversity and distinctiveness of defensive chemical secretions, with more than five new terpenoid alkaloid scaffolds uncovered over the past five years.^12–15^ These new chemicals, some with an unparalleled seven continuous stereogenic centers, arguably are the most unique and complex defensive secretions derived from millipedes, and encompass some of the most surprising advances. This review focuses solely on Colobognatha millipedes: their unique biological characteristics, the chemical composition of their defensive secretions, the biological roles of the terpenoid alkaloids, and insights into a potential biosynthetic pathway.

### Colobognatha Millipedes

Millipedes of the subterclass Colobognatha include the four orders, Platydesmida, Polyzoniida, Siphonocryptida, and Siphonophorida, with eight families and more than 200 described species (Figure 1).^16^ Species of the Colobognatha are found nearly worldwide except north of the Arctic Circle and Antarctica.^17^ Centers of high species diversity are in North America and Europe, with understudied regions such as Asia and Australia likely doubling the known species count. Sampling a 60-m-deep geological borehole in Australia yielded the animal with the most legs—a 1306-legged colobognath millipede, *Eumillipes persephone,* which has uncharacterized alkaloid secretions.^18^ With fossils dating back 99 million years from Cretaceous Burmese amber (*Andrognathus burmiticus*) and molecular clock estimates placing the origin of Colobognatha between 200 and 242 mya, the group has diversified into a wide array of species.^19^ In North America, they are primarily found within the Appalachian, Ozark, and Sierra Nevada mountain ranges; however, there are a handful of observations across the Southeast (*e.g.,* Florida, Alabama, Louisiana, and Texas), the Midwest (*e.g.,* Michigan, Wisconsin, Indiana), and the Southwest (*e.g.,* Arizona). A commonly encountered species of Colobognatha, *Rhinotus purpureus*, has been widely introduced throughout subtropical and tropical regions due to human industry.^20^ Millipedes of the subterclass Colobognatha are mainly found on the underside of decaying logs, in leaf litter, or soil surface at the detritus-soil interface. They possess distinctive morphological features, including pigmentation in hues of pink and purple, small cone-shaped heads, and their bodies can be either cylindrical or flat-backed.

**Figure 1:**
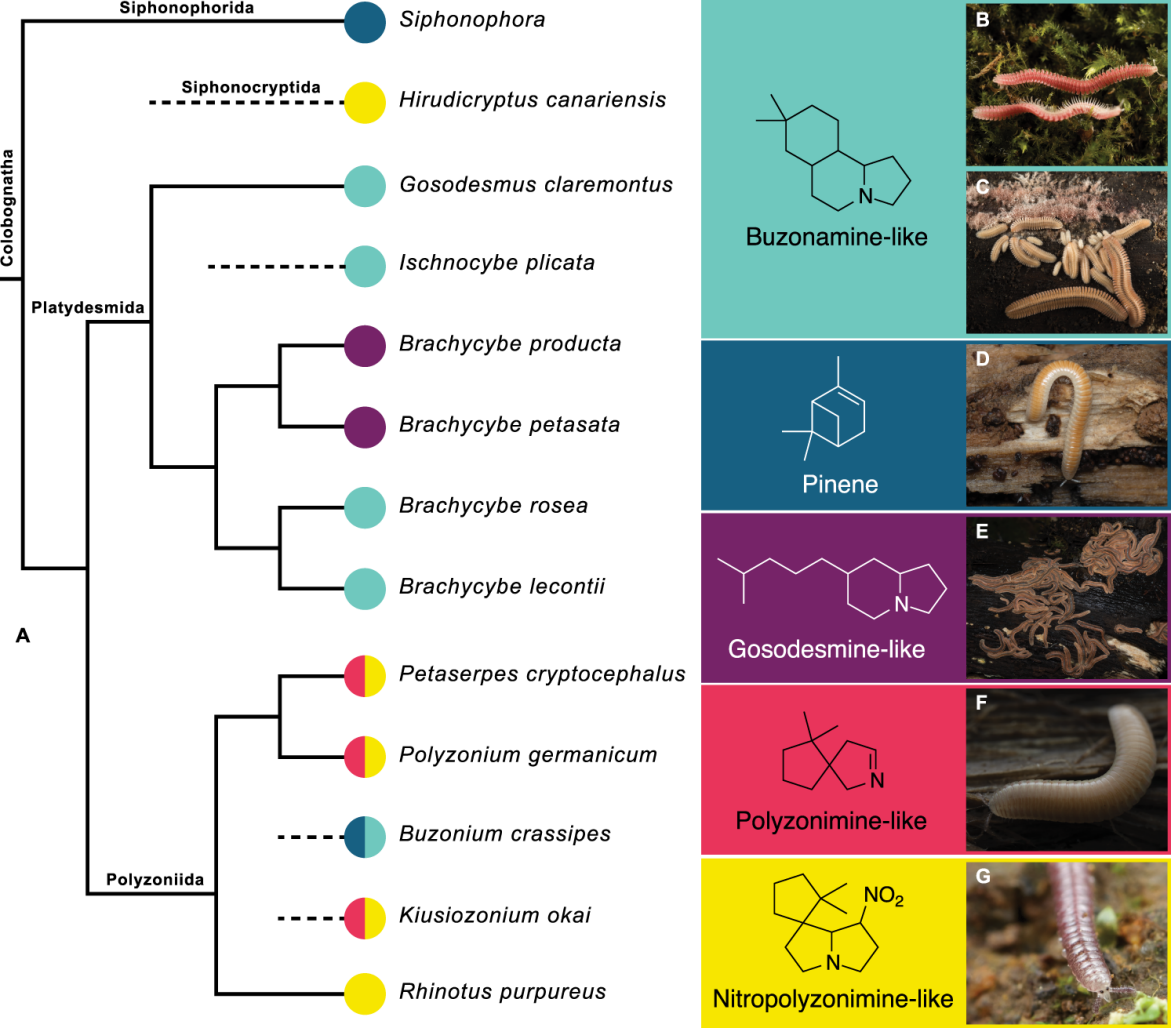
(A) Cladogram depicting the evolutionary relationships of colobognath millipedes with known chemical defense secretions. The types of compounds produced by each millipede are shown.^17,21,22^ (B–F) Pictures of select colobognath millipedes. (B) *Ischnocybe plicata*; (C) *Brachycybe lecontii*; (D) *Buzonium crassipes*; (E) *Brachycybe producta*; (F) *Petaserpes cryptocephalus*; (G) *Rhinotus purpureus*. Consensus cladogram with all compatible groupings represented from refs 16, 18, 21, and 22. The figure was adapted from ref 13. Copyright 2025, ACS Journals.

Colobognath millipedes are a challenging group to study because of their small body size (many are less than 1 mm wide) and their morphologically conserved, primitive-appearing gonopods, which appear largely unchanged across different taxa.^23^ Gonopods are the ninth and 10th leg pairs in Colobognatha that have been modified during development to function as sperm transfer organs in adult males and are traditionally used for species delimitation and classification.^24^ The classification of Colobognatha is outdated, and the monophyly of the group has only recently been supported by molecular phylogenetics.^25,26^ The relationships among its orders are unclear, and the placement of the rare order Siphonocryptida is unknown, but newer genomic data suggest it may be a close relative of Polyzoniida or Platydesmida.^22,27^ Taxa such as *Platydesmus* and *Brachycybe* appear to be close relatives based on recent molecular phylogenetic analyses, but currently belong to different families based on differing morphological characters: Platydesmidae and Andrognathidae, respectively. However, with the significantly reduced cost of DNA and genomic sequencing, there has been a recent focus on clarifying the phylogeny of other nonalkaloid producing millipedes, which has led to revisions of the established phylogeny and current classification.^28^ Similar studies are needed for Colobognatha to improve the current classification system, and an informative evolutionary framework to comprehend the diversity of alkaloid-based chemical defense secretions.

The name Colobognatha originates from the Greek “*colobos*,” meaning reduced and *“gnathos*,” meaning jaw, and derives from the fact that most millipedes within this subterclass have evolved a primitive fluid-feeding, suction method.^29^ This allows them to specialize in consuming fungi as their primary food source. While fluid-feeding is common in insects, only about 2% of living Diplopoda species exhibit this trait.^30^ Although some Colobognatha species seem to prefer certain fungi and are found associated with a single fungus, such as *Ischnocybe plicata* and many *Brachycybe* species, it is generally not believed that they are specialist consumers. However, there is a lack of detailed ecological studies on their dietary preferences. One recent study examined the dietary preference of *Brachycybe lecontii,* which appeared to be associated with highly similar fungi when observed in nature; however, the study concluded that the fungal community found within the gut of *B. lecontii* is usually quite diverse.^31^

### Social Characteristics of Colobognath Millipedes

Colobognath millipedes are known for their distinctive social behaviors, such as forming large aggregations and caring for their young, which set them apart from other millipedes. Due to their fungivory, they are often seen clustered in large groups (up to 100 individuals) on fungi growing on the undersides of decaying logs. These groups typically include millipedes at different developmental life stages, with the exclusion of eggs, and usually consist of a single species. Additionally, several species of Platydesmida, especially those in *Brachycybe* and *Platydesmus*, are frequently seen forming ″pinwheel″ aggregations where all the millipedes face inward toward a central hub and their tails project outward, thereby appearing like the toy.^31,32^ Field observations of various species suggest these millipedes spend part of the year in these aggregations, but become more active in later summer months as pairs of adult millipedes and juveniles were more frequently encountered.^12^ Males appear to adopt a brooding posture, with eggs beneath their curled bodies, and seem to groom the eggs throughout incubation (3–4 weeks), likely to protect them from fungal infections and predators.^33^ Once hatched, the males vacate the natal site with no juvenile millipedes brooded by adults.^32^ Interestingly, many other Colobognatha species also perform egg brood care, a behavior uncommon among other Diplopoda.^4^ Both the formation of aggregations and brood care are rarely seen in other millipede classes, and are hypothesized to require some yet to be identified pheromones.^12,34^

### Defensive Glands and Chemical Composition of Colobognatha

Like most millipedes, Colobognatha have defensive glands, also known as repugnatorial glands, located bilaterally that line the length of the body. Over evolutionary time, glands appeared to diversify into three or four types: (1) bilateral single-chambered glands of Juliformia and Nematophora, (2) median Y-shaped glands of Glomerida, and (3) bilateral bipartite glands of Polydesmida.^4,35^ There are only a few studies of Colobognatha glands. These limited studies show that Polyzoniida and Platydesmida species possess type 1 defensive glands.^4,9^ Initially, the defense glands of *B. lecontii* were described as long, slender tubes, which were proposed to represent a fourth gland morphotype.^9^ However, this was revised when it was shown that the gland architecture of *B. lecontii* is tear-drop-shaped and more voluminous than previously described. The gland of *B. lecontii* consists of a single chamber containing the defense secretions, connected to a duct leading from the ozadene to the ozopore.^32^ The voluminous gland can occupy up to a third of the total volume of a single paranota.^32^ Bipartite and Y-shaped glands are ostensibly derived from a primitive single-chambered gland, but gland morphotypes are known from a limited set of taxa. Ozopores are believed to develop shortly after the millipedes hatch from their eggs, as *B. lecontii* stadia I individuals (∼7 body rings) possess visible ozopores, but no chemical secretions were detected. In stadia II millipedes (14–18 body rings), chemical secretions within the gland were visible, although disturbance did not produce visible secretion. By stadia III (21–30 body rings), secretions were observable internally and externally upon physical disturbances (Table 1).

**Table 1:** Terpenoid Alkaloids Produced by Millipedes of the Subterclass Colobognatha^a^

order	species	natural product	reference
Siphonophorida	*Siphonophora* spp.	α- (**1**) & β-pinene (**2**)*	Shear, 2015
Siphonocryptida	Hirudicryptus canariensis	Spiropyrrolizidine 236 (**6**)*	Shear, 2015
Polyzoniida	Kiusiozonium okai	Polyzonimine (**4**), Nitropolyzonamine (**5**), Spiropyrrolizidine 236 (**6**)	Kuwahara, 2007
	Petaserpes cryptocephalus	Polyzonimine (**4**), Nitropolyzonamine (**5**)	Meinwald, 1975, Smolanoff, 1975
	Polyzonium germanicum	Polyzonimine (**4**), Nitropolyzonamine (**5**), Nitropolyzonamine analogs I–III (**7**–**9**)	Röper, 1978; Kunert, 2023
	Rhinotus purpureus	Nitropolyzonamine (**5**), Spiropyrrolizidine 236 (**6**)	Saporito, 2003
	Buzonium crassipes	β-pinene (**2**), Limonene (**3**), Buzonamine (**10**),	Wood, 2000
Platydesmida	Gosodesmus claremontus	Gosodesmine (**11**)	Hassler, 2020
	Brachycybe producta	Gosodesmine (**11**), Hydrogosodesmine (**12**), Homogosodesmine (**13**), Hydrohomogosodesmine (**14**)	Banks, 2024
	Brachycybe petasata	Gosodesmine (**11**), Hydrogosodesmine (**12**), Homogosodesmine (**13**), Hydrohomogosodesmine (**14**)	Banks, 2024
	Brachycybe lecontii	Deoxybuzonamine 1a (**15**), Deoxybuzonamine 1b (**16**)	Jones, 2022
	Brachycybe rosea	Deoxybuzonamine 1a (**15**)	Banks, 2024
	*Platydesmus* sp.	Unidentified monoterpenes, deoxybuzonamine analog*	Shear, 2015
	Ischnocybe plicata	Unknown Ischnocybine A–C (**17**–**19**), Ischnocybinone (**20**)	Menegatti, 2025
	Andrognathus corticarius	Andrognathines (**21**), Andrognathanols (**22**)	Banks, 2025

a *Those with an asterisk are unconfirmed.

#### Siphonophorida Defensive Secretions

Siphonophorida comprises two families with 116 known species.^36,37^ This order is known to occur predominantly in North and South America (from the southwestern United States through Brazil and Chile), and throughout Southeast Asia, Australia, and New Zealand. It is absent in Europe and most of Africa, spare South Africa and Madagascar. It is believed that species within Siphonophorida produce only simple monoterpenes, such as α- (**1**) and β-pinene (**2**); however, this is based primarily on unpublished studies where the chemical secretions of single species of *Siphonophora* was reported in a review article.^4^

#### Polyzoniida and Siphonocryptida Defensive Secretions

Polyzoniida and Siphonocryptida are two orders within Colobognatha that historically been classified as a single order, but later, Siphonocryptidae was separated as a distinct order based on morphology. However, this relationship should be revisited with genomic sequencing and phylogenomics.^22,27^ The order Polyzoniida is relatively species-rich, with 72 described species, and they have been found on all continents except Antarctica. This order was the first chemically investigated, with compounds described in the 1970s; yet, only six species have been studied: *Kiusiozonium okai*,^38^ *Petaserpes cryptocephalus* (previously known as *Polyzonium rosalbum*),^5,39^ *Polyzonium germanicum*,^15^ *Rhinotus purpureus*,^40^ *Hirudicryptus canariensis*,^4^ and *Buzonium crassipes*.^6^

The first discovery of a terpenoid alkaloid in a millipede’s defensive secretions was in 1975, when polyzonimine (**4**) and nitropolyzonamine (**5**) were identified from the defensive glands of *P. cryptocephalus* (Figure 2).^5,39^ This species is commonly found in eastern North America and can grow up to approximately 18 mm in length. It was observed by Meinwald and colleagues to emit a sticky, whitish discharge when agitated, such as when pinched with forceps.^5^ Before the structures of these alkaloids were elucidated, it was believed that *P. cryptocephalus* secretions only contained camphor due to similarities in texture and odor.^41^ However, analysis of infrared and ^1^H NMR spectra revealed the presence of an imine and a bicyclic system. Total synthesis of the suspected product confirmed the structure of **4**.^5^ Compound **5** was larger and more complex than **4**; however, they managed to elucidate the structure through X-ray crystallography.^39^ Both compounds are spirocyclic alkaloids, with **5** being a spiropyrrolizidine oxime. Analyzing four other Polyzoniida species, including *K. okai*, *P. germanicum*, *R. purpureus*, and *H. canariensis*, revealed that they also produce **4**, **5**, and a related analog, spirropyrrolizidine 236 (**6**).^4,38,40^ Recent reevaluation of the defensive secretions of *P. germanicum* by Kunert et al. uncovered even more chemical diversity that was previously overlooked, including the production of nitropolyzonamine analogs with acylation and oxidation (**7**–**9**).^42–44^ These analogs are present at quantities less than 15% of **4** and were likely missed in earlier studies. Since the discovery of **4**–**6**, there have been several total syntheses of each,^45^ including enantioselective routes,^46,47^ and this established the absolute configurations of **4** and **5.**

**Figure 2:**
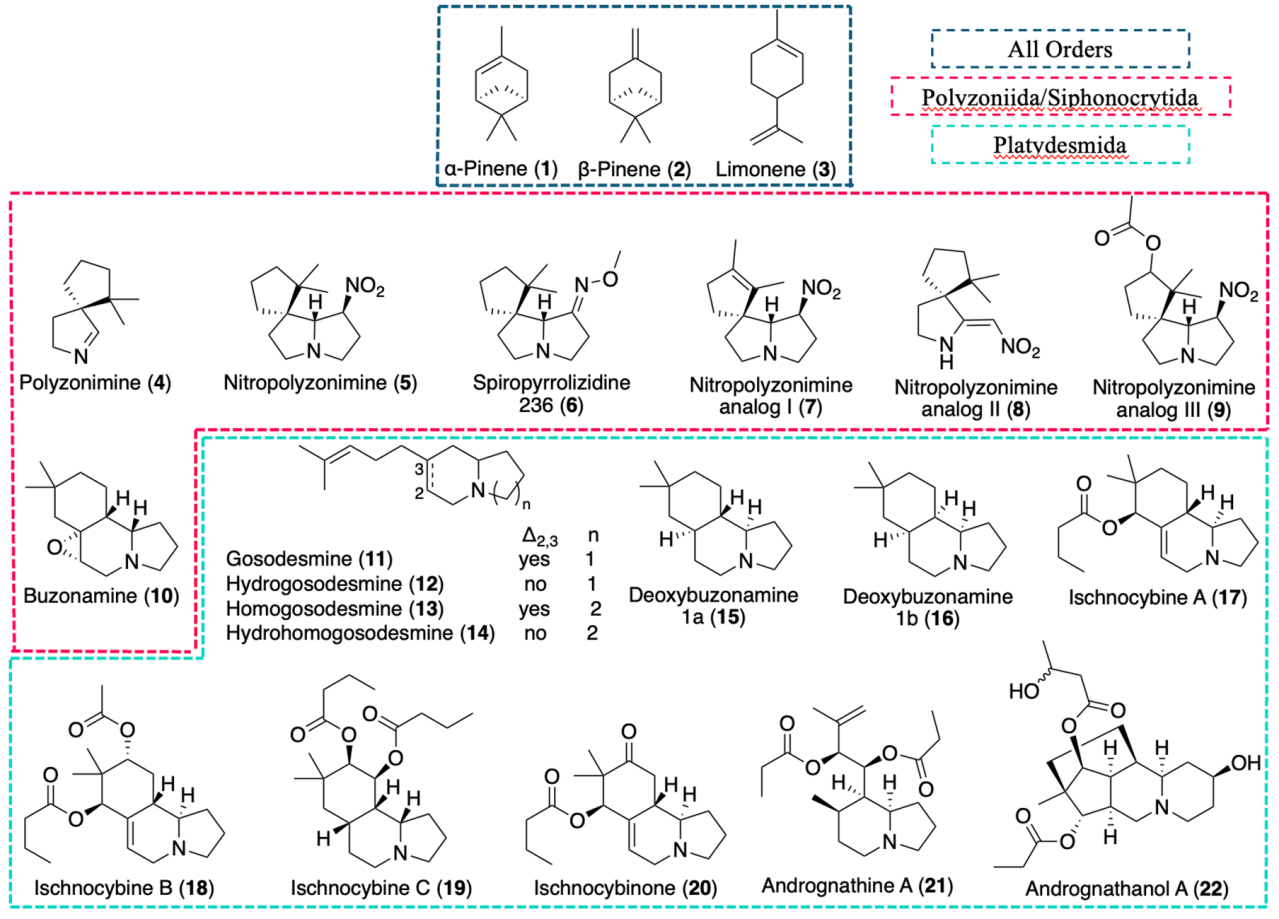
All known Colobognatha-derived natural products, both simple monoterpenes and terpenoid alkaloids.

Interestingly, **6** was first detected, along with structural analogs, in the chemical extracts of poison frogs belonging to the families Dendrobatidae, Mantellidae, and Bufonidae, which are endemic to both Neotropical and Afrotropical regions.^46,48,49^ It is well documented that these frogs consume various arthropods and are capable of sequestering a range of alkaloids on their skins, likely for defense, and this has been the focus of extensive reviews in the past.^40,48–52^ The spiropyrrolizidines were only detected in the skin of the dendrobatid frogs when the frogs were fed fresh leaf litter containing a variety of arthropods, but the alkaloids were absent after feeding the captured amphibians fruit flies or crickets.^46,53,54^ The structural similarity of the spiropyrrolizidines to nitropolyzonamine strongly suggests that co-occurring Colobognatha millipedes that dwell in the leaf litter are the true source of these alkaloids in the amphibian’s skin. Detailed analysis of the frogs’ diet and the defensive secretions of sympatric millipedes confirmed that **6** is produced by polyzoniidan millipedes, specifically *R. purpureus* and *K. okai*.^8^ The source of the other spiropyrrolizidines in the frogs’ diets is unknown, but it is believed to be an as-yet-unstudied polyzoniidan millipede.

To date, the only polyzoniidan millipede species known to produce defensive secretions other than polyzonimine and nitropolyzonamine analogs is *B. crassipes*, which is endemic to the central U.S. Pacific coast. In 2000, Crews and colleagues reported the isolation and structure elucidation of buzonamine (**10**), a new 5,6,6-fused tricyclic alkaloid containing an unusual epoxide functionality.^4,32^ In this case, the defensive secretions from *B. crassipes* were extracted from live millipedes by agitating them on filter paper. This caused the millipedes to discharge their defensive secretions, which were then captured and extracted directly from the filter paper. Quantitative analysis of individual millipedes revealed that each produced approximately 4 mg of secretion, enabling the elucidation of structure through 2D NMR using just 20 millipedes.

#### Platydesmida Defensive Secretions

The order Platydesmida includes two families and 62 species, and are commonly found across North and Central America, Europe, and Southeast Asia.^12,13,34,50^ They are referred to as feather millipedes and often exhibit bright pigmentation that has been suggested to be an aposematic signal.^14^ Until 2020, no chemistry was described from Platydesmida species; however, this situation has changed dramatically over the past five years, with the known chemistry expanding rapidly and leading to the discovery of terpenoid alkaloids with unprecedented structures.^12,13,34,55^ In 2020, Hassler and colleagues reported the discovery of gosodesmine (**11**), a 7-substituted indolizidine alkaloid produced by *Gosodesmus claremontus*, a millipede commonly found in forests of California and Oregon.^3^ Its structure was determined by 2D NMR and was confirmed by total synthesis. An additional indolizidine analog, hydrogosodesmine (**12**), along with two quinolizidines—homogosodesmine (**13**) and homohydrogosodesmine (**14**)—were later identified from the defensive secretions of two *Brachycybe* species: *B. petasata* and *B. producta*.^50^ These structures are distinct from the terpenoid alkaloids characterized from Polyzoniida, as **11**–**14** lack the spirocyclic moiety. *Brachycybe petasata* is endemic to the Appalachian Region, while *B. producta* is endemic to the Central Pacific coast and Sierra Nevada mountains and is sympatric with *G. claremontus*. Genomic sequencing of *B. petasata* and *B. producta* indicates they are sister species that most recently diverged from a common ancestor.^34^

In 2022, analogs of **10** were first discovered in a platydesmidan species. Initially, Jones and colleagues described two new compounds, deoxybuzonamine isomers (**15** and **16**), from the defensive secretions of *Brachycybe lecontii*.^55^ The structures were proposed based on similarities in fragmentation compared to **10,** and this was later confirmed through total synthesis. *Brachycybe lecontii* is endemic to the southeastern United States and partially overlaps in its distribution with *B. petasata*, which produces **11**–**14**. Later, one of these isomers (**16**) was identified as the primary alkaloid in the defensive glands of *Brachycybe rosea*, a sister species to *B. lecontii*, which is endemic to forests along the U.S. Pacific coast.^50^ Interestingly, bulk collections of *B. lecontii* across multiple geographically distinct sites commonly contained both isomers; however, individual collections of millipedes from a single colony contained only a single isomer. This finding led to two hypotheses that the two isomers might mediate signaling conspecifics, such as an aggregation cue or mate signaling.^13^ Alternatively, phylogenetically distinct and geographically limited lineages of *B. lecontii* have been reported, and the presence of isomers **15** or **16** were observed to correlate with membership within an established clade.^55^ However, both of these theories need to be confirmed through chemical ecology studies.

Additionally, an investigation of the secretions of *Ischnocybe plicata*, a millipede found in the U.S. Pacific Northwest, revealed the presence of 5,6,6-fused tricyclic heterocycles that were functionalized through oxidation and ligation.^12^ LCMS and GCMS analysis of chemical extracts from *I. plicata* showed that it produces alkaloids with significantly higher masses than all previously known metabolites (292–360 *m*/*z*), and their fragmentation patterns differ markedly (**17**–**20**). Complete structure determination by 2D NMR, Density Functional Theory (DFT) calculations, Mosher’s esterification, and chiral optical spectroscopy confirmed that the ischnocybines are similar to deoxybuzonamine, but are decorated with various functional groups, including esters derived from short-chain fatty acids and ketones. The compounds were shown to be actively secreted through the ozopores in response to physical disturbances, such as those caused by a glass capillary tube. Finally, quantitative analysis of isolated material indicates that *I. plicata* produces substantial amounts of these alkaloids, approximately 50 μg per millipede, which is consistent with previous reports from Polyzoniida.^5^

Most recently, studies of the secretions of *Andrognathus corticarius* have led to some of the most surprising findings. *Andrognathus corticarius* mainly inhabits the mid- to south regions of the Appalachian Mountains, with some limited observations in Alabama and northern Florida.^8,19,56^ LCMS analysis quickly revealed that *A. corticarius* produces an extensive arsenal of alkaloids (>25 distinct compounds) that differ from all reported metabolites and represent the largest identified to date (241–425 *m*/*z*). Isolation and complete characterization using 2D NMR confirmed the presence of two distinct backbones: the andrognathines (**21**), which contain a 7,8-substituted indolizidine core that differs from that of **11** and **12**, and the andrognathanols (**22**), which feature an unprecedented 6,6,6,5-bridged tetracyclic heterocycle.^53^ The remarkable structural diversity observed in the LCMS chromatogram results from the incorporation of a wide range of different short-chain fatty acids, some of which include hydroxylations or branched chains. However, this chemical diversity within a single millipede species is quite different from all other reports, which contain at most 4–5 individual alkaloids, all within the same structural class. It is currently unknown how this chemical diversity affects the animal’s fitness and ecological interactions.

#### Current Gaps in our Knowledge of Chemical Secretions

Despite the proliferation of reported chemical secretions, fewer than 10% of all described Colobognatha species have been studied, with many genera remaining unexamined. For example, of the 68 described species belonging to Platydesmida, only seven have had their chemistry analyzed, and all are found within the United States. However, Southeast Asia, Central America, and Southern Europe are biodiversity hotspots that have been primarily studied for taxonomy,^57^ with little systematic research into their chemical defensive agents. Millipedes are significantly more diverse closer to the equator; however, taxonomic research on them in these subtropical and tropical regions is limited.^58^ This suggests that the actual number of Diplopoda species is closer to 70,000, with most still undescribed.^5,6,12,13^ Among the described species of Platydesmida, entire genera remain unstudied, including *Platydesmus* and *Desmethus* species common in Central America; *Pseudodesmus* of Southeast Asia; and *Dolistenus, Plutodesmus, Ebenostenus, and Fioria* of the Mediterranean. The genera *Symphyopleurium* and *Yamasinaium* from Japan have also not been investigated. Of the Polyzoniida, 17 genera from biogeographical regions such as Indo-Burma, Mesoamerica, and Japan remain to be analyzed.^57^ Based on our current understanding, chemical investigations into the defensive secretions of millipedes from these hotspots are likely to uncover new chemical compounds and deepen our understanding of their biomedical and ecological roles.

### Ecological and Biological Function Colobognatha Terpenoid Alkaloids

Terpenoid alkaloids are believed to serve a defensive role for the animal. This is supported by the active secretion of these compounds upon disturbance and, in some cases, through behavioral assays involving likely common predators.^5^ However, due to inconsistent types of assays, no comparisons can be made across different structural classes. Polyzonimine (**4**) has been examined most thoroughly, and is the only spirocyclic alkaloid tested in ecologically relevant experiments.^6^ When whole *P. cryptocephalus* were fed to foraging ants in a laboratory enclosure, the millipedes were quickly attacked, but the ants dispersed rapidly, likely due to the release of contents from the defensive glands. This behavior was replicated using synthetic material or crude extracts at ecologically relevant concentrations. The ants’ response to the chemicals, characterized by intensive and prolonged cleaning, led to the conclusion that **4** was likely a general irritant. The level of irritation was measured against both flies and cockroaches, with concentrations as low as 10^–1^ and 10^–4^ M eliciting responses, respectively, which is well within the estimated concentration of the alkaloids within the glands (0.5–1 M).^12,13^ Similarly, assessment of the impact of buzonamine (**10**) on the feeding activities of mound-nesting ants (*Formica obscuripes*) supports a defensive function.^12^ Untreated mealworms are quickly preyed upon when placed outside of the ant mound; however, treatment of the mealworm with either simple monoterpenes or **10** significantly slowed this behavior. Finally, the ischnocybines (**17**–**20**), andrognathine A (**21**), and andrognathanol A (**22**) all impacted the behavior of *Aphaenogaster* sp., a common ant in southwestern Virginia, likely a predator of *A. corticarius*.^54^ Exposure to ecologically relevant concentrations of individual alkaloids or crude extract caused ants to stop moving and induced prolonged cleaning behavior.^46^

However, there is a lack of knowledge about the true predators of colobognath millipedes, and no experimental data are available beyond ants, cockroaches, and flies, which limits our understanding of their fundamental ecological role. The alkaloids may deter predation by a distinct suite of predators, including amphibians (*e.g.,* salamanders, frogs). Studies have shown that poison dart frogs residing in both Madagascar and the Amazon region in South America sequester millipede defenses from their diet.^59^ Upon being brought into captivity, the frogs slowly lose these metabolites, confirming that the amphibians are not producing these metabolites *de novo*.^56^ It is unknown if other amphibians consume the millipedes. Although salamanders are commonly encountered during our millipede collection activities in southwestern Virginia, millipedes and salamanders are rarely found cohabitating under the same decaying logs.

In addition, due to colobognath millipede’s unique social traits (*e.g.,* brood care and sociality), it has been hypothesized that the terpenoid alkaloids may serve a dual function, acting as pheromones as well.^34,60^ This is particularly likely for members of Platydesmida, where there is a significant structural diversity of defensive secretions that correlates with the established phylogeny. For example, most genera produce distinct families of alkaloids, such as *Brachycybe* species producing variants of **11** and **15**, *I. plicata* producing **17**–**20**, and *A. corticarius* producing the **21** and **22**. Preliminary Y-tube choice studies by our laboratories have begun to provide key insights into the potential pheromone role of a subset of the alkaloids. Specifically, millipedes of the same species are significantly attracted to one another, while species from different orders are repelled (unpublished). Although these preliminary results suggest that a subset of the alkaloids behaves as pheromones, much work remains to fully decipher these complex relationships.

The terpenoid alkaloids from colobognath millipedes are reminiscent of other arthropod and amphibian alkaloids that modulate sodium channels (*e.g.,* pumilotoxins and homopumiliotoxins) and are noncompetitive inhibitors of nicotinic acetylcholine receptors (*e.g.,* histrionicotoxins, epiquinamide, gephyrotoxins, and decahydroquinolines).^48^ Only **5**, **6**, and **17**–**20** have been evaluated broadly for neuroreceptor binding activity (Table 2). Compounds **5** and **6** were found to be noncompetitive blockers of the nicotinic receptor with some selectivity for the ganglionic-type [α_3_β_4(5)_], as opposed to the neuromuscular-type (α_1_β_1_γδ).^61^ However, the activity profiles of both **5** and **6** were modest, with IC_50_ values of 1.5 μM. In addition to this activity, both compounds inhibited the binding of the endogenous sigma receptor (σR) ligand with IC_50_ values of approximately 0.5 μM. Compounds **17**–**20** were evaluated more broadly against 53 neuroreceptors through collaboration with the Psychoactive Drug Screening Program (PDSP) at UNC Chapel Hill.^12,57^ Overall, the compounds only potently interacted with sigma-1 receptor (σ_1_R) with good selectivity over the sigma-2 receptor (σ_2_R). Compound **17** exhibited the most potent activity for the σ_1_R with a K_i_ of 13.6 nM, while **18** and **19** were about 30-fold and **20** was 100-fold less active. Interestingly, the free alcohol of **16**, generated through semisynthesis, was significantly less active and lacked selectivity for σ_1_R over σ_2_R. Beyond σR activity, there was also some limited activity observed against the serotonin (5-HT2B), dopamine (D3), histamine (H3), GABAA, and adrenergic (α2C) receptors, but this was observed with only a single compound. Compounds **21** and **22** were only evaluated against σ_1_R and σ_2_R.^58^ Compound **21** exhibited weak activity against σ_1_R with similar selectivity over σ_2_R, while **22** was inactive at concentrations up to 10 μM. Unfortunately, none of the defensive secretions from Platydesmida species have been evaluated in a functional nicotinic receptor assay, which would be required to identify noncompetitive inhibition. Finally, five of the alkaloids (**5**, **6**, **17**, **21**, and **22**) were evaluated against two sodium channels, Na_V_1.5 and Na_V_1.8, but were inactive at concentrations up to 10 μM.^12,61^ This was quite surprising given the activity profile of the highly toxic pumiliotoxins, which are produced by mites and also contain an indolizidine moiety.^62^

**Table 2:** Receptor Activity of the Terpenoid Alkaloids

	compound activity (μM)								
	**5** ^a^	**6** ^a^	**17** ^b^	**18** ^b^	**19** ^b^	**20** ^b^	**21** ^c^	**22** ^c^	**OH-17** ^b^
PC12 [α_3_β4(5)]	1.5	1.5	NT	NT	NT	NT	NT	NT	NT
TE671 (α_1_β_1_γδ)	67	9.5	NT	NT	NT	NT	NT	NT	NT
σRs	0.5	0.5	NT	NT	NT	NT	NT	NT	NT
σ_1_R	NT	NT	0.014	0.38	0.38	1.5	0.84	>10.0	2.2
σ_2_R	NT	NT	1.3	>10.0	2.9	>10.0	2.1	>10.0	2.1
5-HT2B	NT	NT	9.3	5.4	>10.0	>10.0	NT	NT	NT
D3	NT	NT	>10.0	>10.0	>10.0	5.4	NT	NT	NT
H3	NT	NT	>10.0	1.1	>10.0	>10.0	NT	NT	NT
GABAA	NT	NT	>10.0	1.7	>10.0	>10.0	NT	NT	NT
α2C	NT	NT	>10.0	>10.0	>10.0	2.9	NT	NT	NT

a Data from Badio, B., 1996.

b Data from Menegatti, C., 2025.

c Data from Banks, P., 2025. NT = not tested.

### Relationship of Chemical Secretions to Phylogeny

No studies have yet examined the relationship between the composition of chemical defense secretions and the phylogeny of Colobognatha. Therefore, our understanding of the evolution of colobognath chemical defenses is inadequate. However, limited investigations into these relationships within other groups have revealed a stepwise evolutionary increase in the structural complexity of defensive secretions. Within the superfamily Juliformia, oxidized aromatic compounds evolved gradually, with increasing complexity in defensive secretions representing a derived trait.^6^ It was proposed that phenol, the ancestral defensive compound, evolved into benzoquinone, then into methoxy-benzoquinone, and so forth, with many modifications involving oxidation and alkylation.

Analysis of the greater chemical diversity observed across species within Colobognatha has begun to support earlier ideas that chemical diversity aligns closely with phylogeny (Figure 1), but the granularity of this relationship is unknown. Siphonophorida is believed to produce only simple monoterpenes, though this is based on a single analysis reported in a review article and personal conversations.^39^ Sister orders Polyzoniida, Siphonocryptida, and Platydesmida all produce both simple monoterpenes and more complex terpenoid alkaloids. Although monoterpenes are not consistently associated with the chemical secretions of the studied millipedes, monoterpene production is believed to be ubiquitous among these organisms. The omission of monoterpenes from the primary literature is likely due to a focus on more complex natural products, as in all our publications. Therefore, monoterpenes appear to be a symplesiomorphic (shared, primitive) trait within Colobognatha, but more studies are needed to illuminate whether the alkaloids evolved from this trait.

Analysis of the alkaloids within the chemical secretions of Polyzoniida, Siphonocryptida, and Platydesmida provides deeper insights, as their chemical motifs are relatively distinct. The spirocyclic heterocycles (*e.g.,* **4**–**9**) have only been found in Polyzoniida and Siphonocryptida, while most fused heterocycles (*e.g.,* **10**–**22**) are present in Platydesmida. The current hypothesis is that both the spirocyclic and fused heterocycles are apomorphic (derived) traits of their respective orders. Currently, the only known outlier is the production of **10** by *B. crassipes*,^13^ a polyzoniid. *Buzonium crassipes* phylogenetic placement within Polyzoniida is strongly supported by molecular phylogeny (unpublished data), thus suggesting that *B. crassipes* may have gained the ability to produce **10** through heterologous gene transfer or that its production represents convergent evolution. However, additional studies are required to delineate these relationships, particularly studies into the secretion composition of the many unstudied described genera.

### Proposed Biosynthetic Pathway

The known chemistry in Figure 2 is structurally diverse, representing 5,5-spirocyclic to 6,6,6,5-bridged tetracyclic heterocycles that incorporate a range of functionalities, including oxidation, nitration, and ligation. However, nearly all the described alkaloids possess a diagnostic *gem-*dimethyl moiety, which is characteristic of terpenoid biosynthesis. Therefore, all alkaloids are predicted to contain a monoterpene (Figure 3; highlighted in blue), derived from geranyl pyrophosphate (GPP; **23**). Each of the alkaloids contains at least one nitrogen, which is hypothesized to be introduced through the condensation with β-nitropropionic acid (**6**),^39^ pyrrolidine (**10**–**12** and **15**–**21**), or piperidine (**13**, **14**, and **22**).^13^ This condensation product is then predicted to undergo cyclization and postmodification events. Oxidases and ligases are required to produce the nitropolyzonamine analog III (**9**), buzonamine (**10**), ischnocybines (**17**–**20**), andrognathine A (**21**), and andrognathanol A (**22**). However, further studies, such as labeling or bioinformatic analyses, will be needed to confirm these hypotheses and to identify biosynthetic genes.

**Figure 3:**
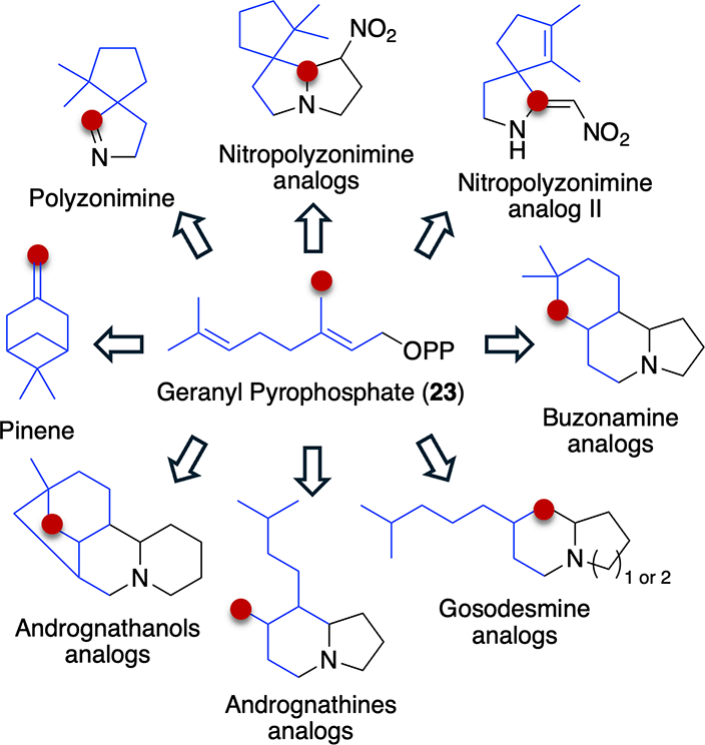
All Colobognatha alkaloids are hypothesized to derive from geranyl pyrophosphate (23; blue), which undergoes different cyclization events, leading to significant structural diversity.

## Summary

In this review, we summarized the current understanding of the chemical diversity and function of the secretions from colobognath millipedes, along with potential future directions. Colobognath millipedes have long been known to produce both simple monoterpenes and alkaloids, which have been shown to deter common insect predators. However, recent discoveries by our group and others have led to an explosion of described chemistry. Since 2020, thirty-one new alkaloids have been identified from species representing two orders (Polyzoniida and Platydesmida). Most of these newly described alkaloids are significantly more complex and include functionalities that were previously unreported within Colobognatha, such as the incorporation of short-chain fatty esters (**9** and **17**–**22**) and additional cyclizations (**22**). In addition to improving our understanding of the secretion composition, these findings offer insights into potential biomedical and ecological functions, while also providing initial glimpses into the evolution of a hypothetical biosynthetic pathway. A subset of the terpenoid alkaloids demonstrates potent affinity for σRs and/or acts as noncompetitive antagonists to nicotinic receptors, indicating potential pharmaceutical applications. Moreover, the increased structural diversity within Platydesmida, where each genus produces distinct secretions, has led to the hypothesis that these compounds serve dual roles—as defensive agents and attractant pheromones. This resurgence has clearly demonstrated that colobognath millipedes are a source of intriguing natural products; however, there is still much to understand about this system. This includes future work into describing the secretion composition of unstudied genera, efforts toward identification of the biosynthetic genes, and understanding their pheromone properties.
